# Voxel-based morphometry in Alzheimers disease and mild cognitive impairment: Systematic review of studies addressing the frontal lobe

**DOI:** 10.1590/S1980-5764-2016DN1002006

**Published:** 2016

**Authors:** Luís Gustavo Ribeiro, Geraldo Busatto

**Affiliations:** 1 BSc, Molecular Sciences Program, Universidade de São Paulo, São Paulo SP, Brazil.; 2 PhD, Department of Psychiatry, Faculdade de Medicina da Universidade de São Paulo, São Paulo SP, Brazil;

**Keywords:** Alzheimer's disease, mild cognitive impairment, voxel-based morphometry, frontal lobe

## Abstract

Voxel-based morphometry (VBM) is a useful approach for investigating neurostructural brain changes in dementia. We systematically reviewed VBM studies of Alzheimer's disease (AD) and mild cognitive impairment (MCI), specifically focusing on grey matter (GM) atrophy in the frontal lobe.

**Methods:**

Two searches were performed on the Pubmed database. A set of exclusion criteria was applied to ensure the selection of only VBM studies that directly investigated GM volume abnormalities in AD and/or MCI patients compared to cognitively normal controls.

**Results:**

From a total of 46 selected articles, 35 VBM studies reported GM volume reductions in the frontal lobe. The frontal subregions, where most of the volume reductions were reported, included the inferior, superior and middle frontal gyri, as well as the anterior cingulate gyrus. We also found studies in which reduced frontal GM was detected in MCI patients who converted to AD. In a minority of studies, correlations between frontal GM volumes and behavioural changes or cognitive deficits in AD patients were investigated, with variable findings.

**Conclusion:**

Results of VBM studies indicate that the frontal lobe should be regarded as an important brain area when investigating GM volume deficits in association with AD. Frontal GM loss might not be a feature specific to late AD only. Future VBM studies involving large AD samples are warranted to further investigate correlations between frontal volume deficits and both cognitive impairment and neuropsychiatric symptoms.

## RESUMO

Morfometria baseada em voxel (MBV) é uma abordagem útil para investigar mudanças neuroestruturais no cérebro em demência. Revisamos sistematicamente estudos de MBV de doença de Alzheimer (DA) e comprometimento cognitivo leve (CCL), focando especificamente na atrofia de matéria cinzenta (MC) no lobo frontal.

**Métodos:**

Duas pesquisas foram realizadas na base de dados do Pubmed. Critérios de exclusão foram utilizados para assegurar a seleção somente de estudos de MBV que investigassem diretamente anormalidades de volume de MC em pacientes com DA e/ou CCL comparados com controles de cognição normal.

**Resultados:**

De um total de 46 artigos selecionados, 35 estudos de MBV reportaram reduções de volume de MC no lobo frontal. As sub-regiões frontais em que a maioria das reduções de volume foram encontradas incluem os giros frontais inferior, superior e médio, bem como o giro do cíngulo anterior. Também acharam-se perdas de MC em pacientes com CCL que desenvolveram DA. Uma menor parte dos estudos investigou correlações entre volumes de MC frontal e mudanças comportamentais ou déficits cognitivos em DA, com achados variáveis.

**Conclusão:**

Os resultados de estudos de MBV indicam que o lobo frontal deve ser visto como uma importante área cerebral quando da investigação de déficits de volume de MC em associação com DA. Perda de MC frontal pode não ser uma característica apenas de DA tardia. Estudos de MBV futuros com grandes amostras de pacientes com DA são necessários para investigar mais a fundo a relação entre déficits de volume frontal e sintomas de comprometimento cognitivo e neuropsiquiátricos.

## INTRODUCTION

Alzheimers disease (AD) is the most common form of dementia, accounting for 50 to 60% of all dementia cases in elderly life.^1^ In 2010, the organisation Alzheimer's Disease International estimated that 35.6 million people were living with dementia worldwide, a figure expected to rise to 65.7 million people by 2030.^2^ Management of the consequences of AD costs governments billions each year. Despite the wealth of scientific discoveries related to AD in the past decades, its causes have not been completely elucidated and further research studies are needed to fully clarify the brain substrate underlying the symptoms of the disorder.

The typical histological and molecular brain pathology that characterises AD leads to structural macroscopic brain changes that can be detected *in vivo* using magnetic resonance imaging (MRI), including most notably atrophy of the grey matter (GM) compartment of the brain.^3^ Such atrophy is thought to begin, and be most prominent, in the temporolimbic hippocampal region.^1^ GM atrophy in AD spreads to other brain regions over the course of the disease, but the patterns of this progression are variable. For instance, it is relevant to elucidate whether (and how) the neuropathological changes associated with AD affect the frontal cortex, since this brain region is critical for short-term memory, attention, planning and motivation.^4^

It is well-known that injuries to the frontal lobe may have multiple deleterious consequences. The prefrontal cortex (PFC) is implicated in several aspects of executive control of behaviour as well as commanding processes that keep concentration and plan-making at optimal levels. Patients with lesions in the dorsolateral portion of the PFC usually commit perseverative errors and lack the ability to choose successful strategies to overcome difficulties. The frontal cortex is also thought to be critically involved in the control of fear, aggression, mating behaviour and other aspects of emotional processing via connections to the amygdala and other limbic structures; more specifically, the orbital ventromedial PFC is thought to mediate appropriate behaviour in stressful situations and correct decision-making in emotionally intense situations.^5^ Impairments in these abilities are either directly detectable in AD patients or underlie the emergence of neuropsychiatric symptoms (such as apathy or agitation); this supports the view that frontal lobe damage is relevant to the pathophysiology of AD. Moreover, a greater understanding about frontal lobe abnormalities in AD is relevant given the need to differentiate this form of dementia from other neurodegenerative disorders which also affect the frontal lobe, such as frontotemporal dementia,^6^ dementia with Lewy bodies^7^ and vascular dementia.^8^

Voxel-based morphometry (VBM) has been developed as an image processing and statistical technique that allows automated, voxelwise investigations of GM volume (GMV) abnormalities in the brain as assessed by MRI and using Statistical Parametric Mapping (SPM) software. One of the relevant features of VBM is its capacity to perform statistical group comparisons of brain volume differences across the whole brain,^9^ rather than on selected regions of interest.

The primary objective of the present systematic review was to evaluate VBM studies that have carried out investigations of GM atrophies located in the frontal lobe of patients with AD or in subjects with mild cognitive impairment (MCI). A secondary objective was to review how previous VBM studies have addressed the cognitive impairments and neuropsychiatric symptoms observed in AD subjects and the possible correlations between these clinical features and GMV reductions in the frontal lobe. 

## METHODS

On September 25^th^, 2015, two searches were carried out on the PubMed database (http://ncbi.nlm.nih.gov/pubmed). The first searched for studies that included the terms "voxel based morphometry AND Alzheimer AND frontal" in their abstracts while the second search encompassed studies which had the following words in their abstracts: "magnetic resonance imaging AND statistical parametric mapping AND Alzheimer AND frontal". Only original articles addressing GMV loss in AD using VBM were included in this review. Studies using non-human subjects and those not written in English were excluded. Studies not comparing AD patients to cognitively normal controls; not analysing the AD group separately from other dementias; and studies which failed to describe the brain regions that showed GMV reductions in AD subjects relative to controls or correlations between GMV and the severity of cognitive and/or psychiatric impairments within the AD group were also excluded.

## RESULTS

The first search led to the retrieval of 106 articles. Although the second search retrieved only 43 articles, it led to the identification of 9 additional VBM studies not identified in the first search. After applying the exclusion criteria described above, a total of 46 VBM studies were selected.

**Figure 1:**
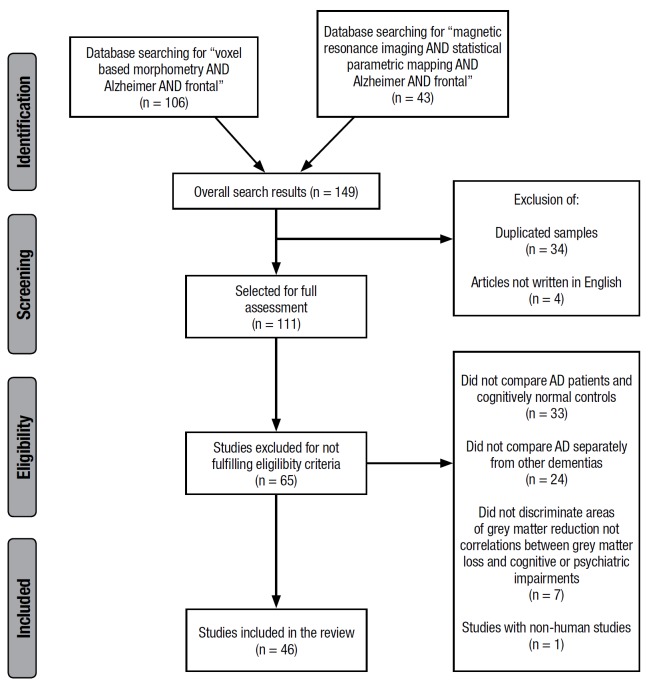
PRISMA flowchart of search results.

Grey matter volume reductions. The AD and MCI groups included in the 46 VBM studies had a mean age ranging from 60 to 82 years. Only 16 studies provided information on disease duration, and this variable ranged from 1.8 years to 6 years, on average, for each group. 

Out of the 46 selected articles, 29 studies reported significant GMV loss and atrophy in the frontal lobe of AD patients, 4 studies reported frontal GMV loss in MCI patients relative to controls, and 2 studies described findings of reduced frontal lobe volume for both AD and MCI groups evaluated separately. Thus, there was a total of 35 VBM studies in which atrophy was described in frontal areas. 

Regarding location, a subtotal of 28 VBM studies of AD noted specific frontal sub-portions involved in the findings of GMV atrophy in AD patients relative to controls, ranked as follows: twelve studies found GMV loss in the right^10^ ^-^ ^21^ and ten in the left inferior frontal gyri,^10^ ^,^ ^11^ ^,^ ^13^ ^-^ ^15^ ^,^ ^18^ ^,^ ^20^ ^-^ ^23^ respectively; seven studies reported GMV reduction in the left middle frontal gyrus,^11,12, 22-26^ left superior frontal gyrus^10^ ^-^ ^12^ ^,^ ^16^ ^,^ ^17^ ^,^ ^27^ ^,^ ^28^ and left anterior cingulate gyrus.^14^ ^,^ ^20^ ^,^ ^21^ ^,^ ^29^ ^-^ ^32^ The right superior frontal^10^ ^,^ ^12^ ^,^ ^16^ ^-^ ^18^ ^,^ ^28^ and anterior cingulate^14^ ^,^ ^20^ ^,^ ^21^ ^,^ ^25^ ^,^ ^29^ ^,^ ^31^ gyri were each noted six times. The right orbitofrontal cortex was mentioned five times;^11, 18,21,26,30^ and there were four mentions of the left^10^ ^,^ ^20^ ^,^ ^21^ ^,^ ^29^ and right medial frontal gyri^10^ ^,^ ^20^ ^,^ ^29^ ^,^ ^30^ as well as the right middle frontal^11^ ^,^ ^20^ ^,^ ^26^ ^,^ ^33^ and left precentral^18^ ^,^ ^28^ ^,^ ^30^ ^,^ ^34^ gyri. Atrophy was found in the right frontal pole^13^ ^,^ ^27^ ^,^ ^35^ and precentral gyrus^18, 28,34^ in three studies each. Finally, GM decreases were also observed in a few studies in cortical pre-motor regions,^28^ ^,^ ^36^ the left frontal pole^27^ ^,^ ^35^ and left orbitofrontal cortex.^21^ The left^21^ ^,^ ^32^ and right^32^ dorsolateral prefrontal cortices were cited in two studies. A few articles described frontal lobe volume abnormalities in AD patients relative to healthy controls in a broad fashion, reporting changes in the overall frontal cortex,^37^ ^-^ ^39^, left prefrontal cortex^40^ ^,^ ^41^ and right prefrontal region.^41^

With regard to MCI, a total of 7 VBM studies searched for GMV loss in the frontal cortex in samples of MCI subjects relative to healthy elderly controls, and six VBM studies found decreases. These changes were reported in the following frontal lobe portions: generally in the frontal cortex^29^ ^,^ ^42^ and anterior regions of the frontal lobe^43^ and specifically in the anterior cingulate gyri bilaterally,^20^ ^,^ ^25^ ^,^ ^44^ besides the right middle frontal^44^ and precentral^44^ gyri. In 5 VBM studies, MCI subjects were followed up in order to ascertain whether they progressed to AD diagnosis during a pre-stablished period of time after the first MRI examination (ranging from 6 to 36 months across separate studies). Among other brain regions, progression to the diagnosis of AD was associated with loss of GM in the following frontal lobe portions: the left superior frontal cortex,^43^ ^,^ ^45^ the bilateral medial frontal gyrus,^20^ ^,^ ^29^ the right superior frontal cortex^43^ and the left inferior^20^ and middle frontal^45^ gyri. Analysing patients more than one year (and up to three years) before conversion, 2 VBM studies found no changes in the frontal lobe.^43^ ^,^ ^46^ The findings for GM loss in MCI and AD patients are shown in Table 1.

**Table 1:** Frontal lobe changes associated with Alzheimer's disease and mild cognitive impairment: results of voxel-based morphometry studies.

Findings	Frontal lobe portions implicated
Alzheimer's disease	
Reduced grey matter volume in comparison to elderly controls (cross-sectional design)	Overall frontal cortex [37, 38, 42] Overall prefrontal cortex [40, 41] Dorsolateral prefrontal cortex [21, 32] Orbitofrontal cortex [11, 18, 21, 26, 30] Frontal pole [13, 27, 35] Inferior frontal gyrus [10-23] Middle frontal gyrus [11, 12, 20, 22, 23-26, 33] Superior frontal gyrus [10-12, 16-18, 27, 28] Medial frontal gyrus [10, 20, 21, 29, 30] Anterior cingulate gyrus [14, 20, 21, 25, 29-32] Cortical pre-motor regions [28, 36] Precentral gyrus [18, 28, 30, 34]
Reduced grey matter volume over time (longitudinal design)	Superior frontal cortex [43, 45] Medial frontal gyrus [20, 29] Inferior frontal gyrus [20] Middle frontal gyrus [45]
Correlation between cortical atrophy and Clinical Dementia Rating scores	Overall frontal lobe [20, 33] Superior frontal gyrus [16]
Correlation between cortical atrophy and Disability Assessment for Dementia scores	Orbitofrontal cortex [37] Precentral gyrus [37] Superior frontal gyrus [37] Inferior frontal gyrus [37] Middle frontal gyrus [37]
Correlation between cortical atrophy and Mini-Mental State Examination Scores	Precentral gyrus [25] Medial frontal gyrus [25]
Correlation between cortical atrophy and episodic memory deficits	Overall frontal lobe [13, 27, 35]
Correlation between cortical atrophy and self-appraisal	Medial prefrontal cortex [41]
Correlation between cortical atrophy and semantic memory deficits	Anterior cingulate cortex [31, 32]
Correlation between frontal cortical atrophy and naming ability	Pre-motor cortex [41] Precentral gyrus [41] Middle superior frontal gyrus [41]
Correlation between cortical atrophy and agitation	Middle frontal cortex [11] Inferior frontal cortex [11] Anterior cingulate gyrus [21]
Correlation between cortical atrophy and depression	Overall frontal cortex [11]
Correlation between cortical atrophy and apathy	Anterior cingulate cortex [12] Orbitofrontal cortex [21] Dorsolateral prefrontal cortex [21]
Correlation between cortical atrophy and aberrant motor behaviour	Olfactory gyrus [11] Medial orbitofrontal gyrus [11] Inferior frontal gyrus[11] Middle frontal gyrus [11]
Amnestic mild cognitive impairment	
Reduced grey matter volume in comparison to elderly controls (cross-sectional design)	Overall frontal cortex [29, 42] Anterior regions of frontal lobe [43] Anterior cingulate gyrus [20, 25, 44] Middle frontal gyrus [44] Precentral gyrus [44]

Correlations between VBM findings and overall severity of dementia in Alzheimer's disease subjects. Five VBM studies investigated whether GMV loss in the frontal lobe was related to the overall severity of dementia in AD patients.^16^ ^,^ ^20^ ^,^ ^26^ ^,^ ^33^ ^,^ ^37^

Three of the cited studies used the Clinical Dementia Rating (CDR) scale.^16^ ^,^ ^20^ ^,^ ^33^ One of these studies divided the group of AD patients according to their CDR scores. While both patients with CDR scores of 1 or 2 presented global GM volume reductions, a greater degree of frontal GM volume deficits was seen in AD patients with more severe forms of dementia (CDR of 2), affecting mainly the bilateral superior frontal gyrus.^16^ Two other VBM studies showed that the severity of CDR scores was directly related to GMV reductions in the frontal lobe of AD patients.^20^ ^,^ ^33^

Two VBM studies focused on the ability to perform daily activities. One of these used the Functional Activities Questionnaire (FAQ), and the authors reported no significant correlations between higher FAQ scores and loss of GM in the frontal lobe.^26^ The second study used the Disability Assessment for Dementia (ADLs) and found significant correlations between: poorer sub-scores for basic ADLs and reduced volume of the left superior frontal and precentral gyri as well as the left orbitofrontal cortex; and worse instrumental ADL's sub-scores and atrophy in bilateral inferior, right superior, and right middle frontal gyri.^37^

Finally, another interesting VBM study followed up subjects with the amnestic subtype of MCI by serial MRI acquisitions over 3 years in order to map the progression of cerebral atrophy towards the full-blown diagnosis of AD.^26^ The clinical deterioration of patients was documented by worsening of CDR scores. While MCI subjects presented GM loss restricted to temporolimbic structures 3 years before the diagnosis of AD, reduced GM volume was widespread in the brain 3 years later when CDR scores were more severe and the diagnosis of AD was confirmed, and GM atrophy substantially affected the frontal lobes for the first time.

Correlations between VBM findings and cognitive deficits. Fourteen studies explicitly investigated whether the frontal GMV loss was related or otherwise to patients' cognitive status, by estimating the significance of statistical correlations between GMV reductions and severity of cognitive deficits in AD and MCI subjects, as assessed by standardised tests at the time of MRI scanning. 

Two VBM studies investigated the relationship between GMV in the frontal lobe and Mini-Mental State Examination (MMSE) scores in AD patients.^14^ ^,^ ^25^ Using strict statistical criteria of significance corrected for multiple comparisons, neither of these 2 studies reported significant findings in the frontal lobe. However, one of them reported a weak inverse association between MMSE scores and GMV of the right precentral and left medial frontal gyri with statistical tests uncorrected for multiple comparisons.^25^

Seven of the above 14 studies addressed memory impairments, documenting episodic memory loss by testing the patients' immediate and delayed recall of sentences and/or pictures. Three studies included the frontal lobes bilaterally as brain areas significantly correlated to episodic memory loss,^13^ ^,^ ^27^ ^,^ ^35^ whilst three other VBM studies found no correlation of memory performance with volume of the frontal lobe.^23^ ^,^ ^30^ ^,^ ^47^ One study used the DemTect protocol to examine memory impairments and language performance of AD patients, but also failed to detect any significant correlation between frontal lobe volume deficits and poorer cognitive scores.^40^

The presence of semantic confabulations, defined as false narratives invented by patients to cover up for memory deficits, were investigated in only one VBM study. The authors found that the severity of confabulations was significantly related to GMV deficits in the anterior cingulate gyrus in AD patients.^31^

A significant association was found in one VBM study between GMV and self-appraisal (the ability one has to judge and determine his/her own physical or mental capacities) in the medial prefrontal cortex in AD patients.^41^ Two other VBM studies detected significant associations between GMV and naming performances. In one of these studies, subjects were asked to name known sounds^38^ and performance on this task was correlated with GMV in the cortical pre-motor regions and precentral gyrus bilaterally, as well as in the middle superior frontal gyrus. In the other of these 2 VBM studies, subjects' semantic retrieval and visual functioning was tested^32^ and the performance was associated with GMV mainly in the temporal lobe, but also in the left anterior superior cingulate cortex. 

Correlations between VBM findings and neuropsychiatric symptoms. Only six VBM studies investigated whether or not there were significant correlations between frontal GMV loss and the severity of neuropsychiatric symptoms associated with AD. Four of these used the Neuropsychiatric Inventory (NPI), or derivations of it, to evaluate the severity of neuropsychiatric manifestations. Derivations included the NPI Questionnaire (a simplified version of the NPI (in which symptoms are described as present or absent and severity is measured in a scale from 0 to 3) and the NPI-12 (in which severity and frequency are scored on a scale from 0 to 12 for each symptom).^11^ ^,^ ^21^ ^,^ ^35^ ^,^ ^44^ One study evaluated AD patients presenting depression, as assessed by the Geriatric Depression Scale (patients with scores greater than 20 were considered depressed),^12^ whilst another study assessed the presence of confabulations (based on reports by caregivers) using the subcategory A of the Behavioural Pathology in AD Frequency Weighted Severity Scale.^31^

One VBM study evaluated solely overall NPI ratings, and reported significant negative correlations between NPI scores in AD patients and GMV in the bilateral middle frontal gyri and right orbitofrontal cortex.^26^

With regard to sub-symptom NPI scores, one study found atrophy in the anterior cingulate gyrus, right middle frontal gyrus, precentral gyrus, and left orbitofrontal cortex to be inversely correlated with the severity of NPI-based disinhibition scores in AD subjects.^44^

Two VBM studies addressed the relationship between frontal GMV with the severity of delusions, as assessed by the NPI. One simply reported atrophy in the overall frontal cortex in subjects with an NPI sub-score indicating delusions,^26^ while the other reported reduced GMV in the bilateral inferior frontal gyri, left medial frontal gyrus^43^ in association with the presence of delusions. A further VBM study reported no significant association between a neuropsychiatric "episodic" type of confabulation involving delusions and GMV in the frontal lobes.^31^

The degree of agitation or apathy, as assessed by the NPI, was found to show significant correlations with frontal GMV in 2 VBM studies. Agitation was correlated with reduced GMV in the left middle and inferior frontal cortices^11^ and the anterior cingulate gyrus.^21^ In regard to apathy, significant negative correlations were reported with atrophy in the anterior cingulate cortex^11^ and with reductions in the bilateral orbitofrontal and dorsolateral prefrontal cortices.^21^

In one study, there was a general link between the severity of depression and GM loss in the left frontal cortex.^11^ Another study found no significant correlations between the presence of depression and GMV in the frontal lobe, but found significant correlation in the temporal lobe.^12^

Finally, aberrant motor behaviour assessed with the NPI was negatively correlated with GMV in the right olfactory, right medial orbitofrontal, right inferior, and right middle frontal gyri in one VBM study.^11^ All findings regarding correlations between atrophy and cognitive or psychiatric deficits are given in Table 1. 

## DISCUSSION

This systematic review of VBM studies addressed the existence of GMV reductions in frontal regions of patients with AD and the relationship of these GMV deficits with cognitive and neuropsychiatric consequences of the disease. The findings reported in this review clearly show that VBM studies have demonstrated AD-related GM atrophy in the frontal lobe - we identified more than 30 studies that have reported GMV abnormalities located in the frontal lobe in association with the diagnosis of AD or MCI. This indicates that the frontal lobe should be regarded as an important brain area when investigating GMV deficits in association with the diagnosis of AD. 

Those VBM studies which reported positive findings indicated that GM atrophy in AD was widespread throughout the frontal lobe, since several frontal subregions were implicated in this literature. Nevertheless, there are frontal subregions that seem to be preferentially affected in AD, namely the superior-lateral and medial prefrontal regions of the frontal lobe, including the bilateral inferior, middle, medial and superior frontal gyri. Thus, despite methodological differences between VBM studies, there seems to be convergence in regard to the preferential location of frontal GMV deficits in association with AD. This pattern of results provides a map indicating the frontal subregions that may be most critically involved in the pathophysiology of AD. 

Previous literature findings indicate that the process of neurodegeneration in AD starts in medial temporal regions, involving mainly the hippocampal/amygdala complex and the entorhinal cortex.^1^ According to this view, atrophy of the frontal cortex would only be expected at later disease stages of AD. Only a minority of the VBM studies reviewed provided information regarding disease duration. However, it is notable that some of the AD samples investigated had a disease duration of 3 years or less, and findings of GMV loss was indeed detected in the frontal lobe in these studies.^13^ ^,^ ^19^ ^,^ ^38^ Moreover, we also found studies in which reduced frontal GM was detected in MCI patients who converted to AD.^20^ ^,^ ^29^ ^,^ ^43^ ^,^ ^45^ ^,^ ^46^ These VBM findings suggest that AD-related frontal GM volume loss might be present at earlier stages of AD, and therefore should not be considered a feature specific to late AD only. 

Given the progressive neurodegenerative course of AD, frontal lobe volume changes in VBM studies will likely become more severe over time in AD patients. Although this issue was not investigated directly in the current study, it is noteworthy that there is an apparent tendency among the patterns of frontal lobe GM decrease; the VBM studies including patients with greater disease duration in general seemed to show more widespread areas of frontal lobe atrophy,^18^ ^,^ ^37^ ^,^ ^41^ often detecting GMV loss in multiple frontal gyri. Conversely, studies evaluating AD samples with more recent disease onset appeared to detect less widespread patterns of GM atrophy, located in more specific frontal lobe sites.^19^ ^,^ ^38^

Another important finding of the present review was the degree of variability of findings regarding the relationship between frontal lobe GMV deficits in AD patients and the severity of cognitive decline. One possible explanation is the fact that episodic memory and other key cognitive features require the involvement of large-scale networks that implicate several other brain regions besides the frontal cortex.^13^

Our systematic review also showed that the number of VBM studies investigating the relationship between frontal GMV and neuropsychiatric symptoms to date has been lower than the number of studies evaluating cognitive deficits in AD. This may be explained by the greater emphasis given to memory loss in AD, although the importance of behavioural changes and psychiatric symptoms associated with AD is now being recognised. The findings of our review suggest that frontal lobe volume deficits may be directly related to various neuropsychiatric manifestations in AD patients. Thus, the relationship between behavioural changes and frontal GMV is likely to be a topic of particular relevance for future VBM studies with larger AD and MCI samples. 

Among the 46 VBM articles selected, some reported no GMV reduction in frontal areas.^48^ ^-^ ^52^ As mentioned above, there were also some discrepancies in terms of the correlations between cognitive variables and GMV loss in the VBM studies included in this review. Methodological issues may have contributed to those discrepant findings. For instance, a significant proportion of VBM studies did not report the duration of dementia of AD samples at the time of MRI scans. Some of the VBM studies may have included AD patients with very recent onset, and these AD patients would presumably present more restricted areas of GMV reduction; depending on the size of the AD samples included in each study and how strict the statistical correction for multiple comparisons, findings in the frontal lobe might not be detectable in some studies involving AD patients with recent disease onset. In fact, it is important to highlight that the VBM studies selected in this review varied in regard to their methods of statistical inference; while some did not use correction for multiple comparisons, others used familywise error methods, and a number of other studies chose to employ false discovery rate corrections. These variations imply that findings in the frontal lobe might have been minor or discarded in VBM studies that chose to use stricter statistical correction for multiple comparisons. Moreover, it is important to point out that the AD groups included also differed across studies for other characteristics such as mean age, education, sex ratio, and genotype (presence or absence of apolipoprotein E-e4, for example).

It should be noted that the use of other biomarkers to reinforce the diagnosis of AD or neuropathological confirmation occurred in a minority of the neuroimaging investigations (only eight studies) reviewed. Four investigations included b-amyloid peptide or protein tau measurements in cerebrospinal fluid (CSF),^14^ ^,^ ^23^ ^,^ ^38^ ^,^ ^41^ one performed positron emission tomography imaging with Pittsburgh compound B (^11^C-PIB PET) to map b-amyloid peptide deposition in the brain^34^ and three studies conducted *post-mortem* confirmation of AD neuropathology.^36^ ^,^ ^51^ This is an important limitation; without the use of AD-related biomarkers or *post-mortem* confirmation, one cannot rule out the possibility that subjects suffering from other neurodegenerative diseases, such as frontotemporal dementia, were inadvertently included in the AD groups examined in VBM investigations.^56^

Finally, the methodological limitations of the present review should also be highlighted. First, it should be noted that some of the selected articles used MRI sets from previously acquired data banks, and several different VBM studies drew on MRI datasets from the same institutions. For instance, both Fischer et al.^46^ and Hu et al.^11^ used the Alzheimer's Disease Neuroimaging Initiative (ADNI) datasets, hence creating the possibility of the same subjects being evaluated by more than one group of authors cited in the present review. Another limitation pertains to our decision to include the word "frontal" among the terms used in our PubMed searches, where this may have biased the results towards positive findings, selecting articles that necessarily mentioned this cerebral region in the abstracts of their articles. By including this search term, we may have VBM studies that investigated GMV deficits in AD patients across the entire brain but did not mention the frontal cortex in their abstracts, conceivably due to a lack of significant findings in this brain region. Despite this limitation, the methodology chosen identified a relatively large number of relevant VBM studies, thus allowing us to fulfil the aim of demonstrating that the VBM technique detects frontal GMV deficits in AD samples. The VBM studies reviewed also indicated those frontal lobe sub-portions most often affected in AD, and provided preliminary information on the relationship between frontal GMV deficits and both cognitive impairment and neuropsychiatric symptoms related to AD. More comprehensive systematic reviews and meta-analyses are warranted to further clarify these highly relevant issues.
